# Two Putative Polysaccharide Deacetylases Are Required for Osmotic Stability and Cell Shape Maintenance in Bacillus anthracis*[Fn FN2]

**DOI:** 10.1074/jbc.M115.640029

**Published:** 2015-03-30

**Authors:** Sofia Arnaouteli, Petros Giastas, Athina Andreou, Mary Tzanodaskalaki, Christine Aldridge, Socrates J. Tzartos, Waldemar Vollmer, Elias Eliopoulos, Vassilis Bouriotis

**Affiliations:** From the ‡ Department of Biology, Enzyme Biotechnology Group, University of Crete, Vasilika Vouton, 70013 Heraklion, Crete, Greece,; the § Department of Neurobiology, Hellenic Pasteur Institute, Vasilissis Sofias 127, 11521 Athens, Greece,; the ¶ Department of Biotechnology, Laboratory of Genetics, Agricultural University of Athens, Iera Odos 75, 11855 Athens, Greece,; the ‖ Institute of Molecular Biology and Biotechnology, 70013 Heraklion, Crete, Greece,; the ** Institute for Cell and Molecular Biosciences, Centre for Bacterial Cell Biology, Newcastle University, NE2 4AX Newcastle upon Tyne, United Kingdom, and; the ‡‡ Department of Pharmacy, University of Patras, 26504, Patras, Greece

**Keywords:** cell wall, Gram-positive bacteria, lipoprotein, membrane protein, x-ray crystallography, Bacillus anthracis, cell shape, osmotic stability, polysaccharide deacetylases

## Abstract

Membrane-anchored lipoproteins have a broad range of functions and play key roles in several cellular processes in Gram-positive bacteria. BA0330 and BA0331 are the only lipoproteins among the 11 known or putative polysaccharide deacetylases of *Bacillus anthracis*. We found that both lipoproteins exhibit unique characteristics. BA0330 and BA0331 interact with peptidoglycan, and BA0330 is important for the adaptation of the bacterium to grow in the presence of a high concentration of salt, whereas BA0331 contributes to the maintenance of a uniform cell shape. They appear not to alter the peptidoglycan structure and do not contribute to lysozyme resistance. The high resolution x-ray structure of BA0330 revealed a C-terminal domain with the typical fold of a carbohydrate esterase 4 and an N-terminal domain unique for this family, composed of a two-layered (4 + 3) β-sandwich with structural similarity to fibronectin type 3 domains. Our data suggest that BA0330 and BA0331 have a structural role in stabilizing the cell wall of *B. anthracis*.

## Teaser

**Background:** BA0330 and BA0331 are the only two lipoproteins among 11 known or putative polysaccharide deacetylases from *Bacillus anthracis.*

**Results:** Both proteins lack deacetylase activity and are important for cell shape maintenance (BA0331) or high salt stress adaptation of the bacterium (BA0330).

**Conclusion:** BA0330 and BA0331 stabilize the cell wall of *B. anthracis*.

**Significance:** Understanding the mechanisms by which lipoproteins maintain cell wall integrity.

## Introduction

*Bacillus anthracis* is a Gram-positive, spore-forming bacterium and is the etiological agent of anthrax, a lethal disease sporadically affecting humans and animals. Its genome is similar to that of *Bacillus cereus*, although both pathogens occupy different ecological niches. The cell wall of *B. anthracis* is composed of peptidoglycan (PG),^3^ polysaccharides, proteins, and a poly-γ-d-glutamic acid capsule (1). PG is a heteropolymer made of GlcNAc and *N*-acetylmuramic acid containing glycan chains cross-linked by short peptides. The cell wall is the stress-bearing structure protecting the cell against lysis due to the osmotic pressure, therefore maintaining cell shape; and enzymes involved in synthesis, remodeling, and turnover of PG play crucial roles in cell growth and division (2).

Gram-positive bacteria have developed mechanisms for retaining proteins within their cell envelope, including covalent linkage to PG and noncovalent binding to teichoic acids and other cell envelope polymers (3–5) and membrane anchoring by N-terminal lipidation of proteins (6, 7). Bacterial lipoproteins are a functionally diverse class of peripheral membrane proteins in Gram-positive bacteria, with important roles in substrate binding for ABC transporters, adhesion, antibiotic, lantibiotic and bacteriocin resistance and phage superinfection, cell envelope homeostasis, protein secretion, folding and localization, redox and sensory processes, including signaling in sporulation and germination (8). *B. anthracis* is predicted from genomic sequence analysis to have a large number of cell-associated lipoproteins, composing ∼2.5% of its proteome (9).

Some Gram-negative bacteria covalently anchor an abundant outer membrane lipoprotein to PG to stabilize the cell envelope by forming tight connections between PG and the outer membrane (6). For Gram-positive bacteria, which lack an outer membrane, structural interactions between lipoproteins and PG have not yet been reported (7).

Polysaccharide deacetylases (PDAs) belong to carbohydrate esterase family 4 (CE4), which includes chitin deacetylases, acetylxylan esterases, xylanases, rhizobial NodB chitooligosaccharide deacetylases, and PG deacetylases. Members of this family catalyze the hydrolysis of either the *N*-linked acetyl group from GlcNAc residues (chitin deacetylase, NodB, and peptidoglycan GlcNAc deacetylase) (10, 11) or *O*-linked acetyl groups from *O*-acetylxylose residues (acetylxylan esterase, and xylanase) (12–14). Interestingly, the genomes of *Bacillus* sp., and especially of *B. cereus* sensu lato, including *B. anthracis,* contain multiple putative polysaccharide deacetylase genes with high sequence homologies. The physiological role of five PDAs in *B. anthracis* has been recently elucidated. BA1977 associated with lateral PG synthesis is the only deacetylase involved in resistance to host lysozyme and is required for full virulence. BA1961 and BA3679 deacetylate PG during both cell division and elongation, whereas BA5436 and BA2944 are important for PG attachment of neutral polysaccharide, which anchors S-layer proteins, and for polysaccharide modification, respectively (15).

The structures of CE4 enzymes from various bacterial species have been determined, including PG deacetylases from *Streptococcus pneumoniae* (16) and *Bacillus subtilis* (17), acetylxylan esterases from *Clostridium thermocellum* and *Streptomyces lividans* (18), poly-β-1,6-*N*-acetyl-d-glucosamine deacetylase from *Escherichia coli* (19) and *Staphylococcus epidermidis* (20), and putative PDAs from *B. anthracis* (21, 22), *B. cereus* (23), and *Streptococcus mutans* (24). CE4 enzymes contain a conserved NodB homology domain and adopt a (α/β)_8_ barrel fold. Most of the structures contain a divalent ion in the active site bound in a His-His-Asp triad. The catalytic machinery is completed by an aspartic acid and a histidine that act as the catalytic base and catalytic acid, respectively (16). Five conserved sequence motifs are required for activity of the CE4 NodB domain. The first Asp residue of motif 1 (TFDD) is believed to act as the catalytic base, which activates the catalytic water, and the second Asp coordinates the metal ion. motif 2 (H(S/T)*XX*H) contributes the two His residues, which coordinate the metal. motif 3 (RpP*X*G) contributes a conserved Arg residue that coordinates the catalytic base and a strictly conserved Pro residue. The catalytic acid is a His residue, which lies in motif 5 and is coordinated by an Asp residue provided by motif 4 (16).

BA0330 and BA0331 from *B. anthracis* are predicted as lipoproteins and putative PDAs and share 55% sequence identity. Furthermore, BA0330 shares 91% identity with its corresponding homologue BC0361 from *B. cereus*, whereas a homologue of BA0331, which is present in all *B. anthracis* strains, is missing in many *B. cereus* strains, including *B. cereus* ATCC 14579. BA0331 is mainly expressed during the exponential phase but is secreted at lower amounts during the stationary phase, in both the avirulent *B. anthracis* UM23C1-2 (pXO1- and pXO2-) and the wild-type virulent Vollum strain (25, 26).

In this study, we employed biochemical and genetic (knock-out) analysis, structure determination, and protein localization to elucidate the biological roles of BA0330 and BA0331 from the avirulent *B. anthracis* UM23C1-2 strain. We show that BA0330 and BA0331 interact with PG and stabilize the cell wall of *B. anthracis*. Furthermore, BA0331 is important for maintenance of the cell shape, and BA0330 contributes to the adaptation of the bacterium to high salt stress. The structure of BA0330 reveals a differentiation of the catalytic machinery of PDAs and could explain the apparent lack of activity of this protein. To our knowledge, this is the first report of lipoproteins implicated in the maintenance of cell wall integrity in Gram-positive bacteria.

## Experimental Procedures

### 

#### 

##### Materials

Primers were synthesized by the Microchemistry Facility of the Institute of Molecular Biology and Biotechnology (supplemental Table S1). The strains and plasmids used in this study are listed in Table 1. All chromatographic materials were from Amersham Biosciences. PCR and gel extraction kits were from Qiagen, and the plasmid purification kit was from Macherey Nagel GmbH. Substrates and common reagents were purchased from Sigma, Seikagaku Corp., and Merck. Fluorescamine and brain-heart infusion (BHI) were purchased from Sigma. Anti-GFP rabbit serum (polyclonal antibody) was purchased from Molecular Probes.

**TABLE 1:** **Strains and plasmids used in this study**

Strains, plasmids	Description	Source or Ref.
**Strains**		
*E. coli*		
DH5α	*F^−^ Φ80lacZΔM15 Δ* (*lacZYA-argF*) *U169 recA1 endA1*	Novagen
	*hsdR17* (*rK^−^, mK^+^*) *phoA supE44 λ^−^ thi-1 gyrA96 relA1*	
BL21 DE3 (pLYS)	*F*^−^ *ompT gal dcm lon hsdS_B_*(*r_B_*^−^ *m_B_*^−^) λ(*DE3*) *pLysS*(*cm^R^*)	Novagen
GM48	*thr-1*, *araC14*, *leuB6*(Am), *fhuA31*, *lacY1*, *tsx-78*, *glnX44*(AS), *galK2*(Oc), *galT22*, λ^−^, *dcm-6*, *dam-3*, *thiE1*	Coli Genetic Stock Center (CGSC)
*B. anthracis*		
UM23C1–2	pXO1- pXO2- Ura- Rifr	66

**Plasmids**		
pGEM T-easy	Cloning vector	Promega
pUTE583	Cloning vector	35
pREST A	Cloning vector	Novagen
pHW1520	Cloning vector	Mobitec
pNF8	pAT18Ω (PdltΩgfp-mut1)	37
pSPCH+1 + 2 +3	pUC19 carrying a nonpolar mutagenic SpcR cassette	34

##### Cloning and Expression of ba0330 and ba0331 Genes of B. anthracis into pRSET A Expression Vector

The genes were amplified from genomic DNA of *B. anthracis* UM23C1-2 using DNA polymerase chain reaction. Primers were synthesized to exclude the signal peptide (1–23 amino acids) and to incorporate a blunt end at the start and an XhoI site at the end of *ba0330* and *ba0331* genes. The amplified genes were purified, digested with the corresponding enzymes, and ligated into pRSET A vector. The resulting products were in-frame, non-His_6_ tag-fused constructs in pRSET A for *ba0330* and *ba0331* genes, placing the PDA genes under the transcriptional control of the T7 *lac* promoter. The two constructs were transformed into BL21(DE3) (pLys) *E. coli* strains. Twenty milliliters of saturated culture of each of the transformed deacetylase expression strains were inoculated into 1 liter of Luria-Bertani (LB) medium containing 100 μg ml^−1^ ampicillin and 34 μg ml^−1^ chloramphenicol as antibiotics and incubated at 37 °C on a shaker incubator to an *A*_600_ of 0.6. BA0330 *E. coli* culture was transferred to 20 °C after addition of 0.5 mm isopropyl β-d-thiogalactoside, and BA0331 *E. coli* culture was transferred to 30 °C after addition of 0.5 mm isopropyl β-d-thiogalactoside.

##### Purification of Recombinant BA0330 and BA0331

For BA0330, the cells were harvested by centrifugation and resuspended in 50 mm Tris-Cl buffer, pH 7.6, 300 mm NaCl, 1 mm dithiothreitol, and 0.3 mg ml^−1^ lysozyme. After 150 min of incubation at 4 °C, the suspension was centrifuged, and soluble fractions were collected and loaded onto an SP-Sepharose Fast Flow chromatography column equilibrated with 50 mm HEPES-NaOH, pH 6.8. Proteins were eluted using a step gradient of NaCl (500 mm). Fractions containing BA0330 were collected and loaded onto a Sephacryl S-200 HR column equilibrated with 50 mm Tris-HCl, pH 7.6, 300 mm NaCl. Fractions containing BA0330 were collected, concentrated, and stored at 4 °C.

For BA0331, all steps prior to chromatographic purification of BA0331 were the same as described previously for BA0330. Subsequently, soluble fractions containing BA0331 were loaded onto a Source Q HR adsorbent equilibrated with 20 mm Tris-HCl, pH 8.5. Proteins were eluted using a step gradient of NaCl (300 mm). Fractions containing BA0331 were collected and loaded onto a Sephacryl S-200 HR column equilibrated with 50 mm Tris-HCl, pH 7.6, 300 mm NaCl. Fractions containing BA0331 were collected, concentrated, and stored at 4 °C.

##### Data Collection, Structure Solution, and Refinement

BA0330 crystallization screening was set up using an Oryx4 crystallization robot (Douglas Instruments Ltd.) with the vapor diffusion method in sitting drops. Protein crystals appeared only in one out of ∼700 conditions tested and were subsequently optimized to produce crystals suitable for diffraction experiments. The best crystals were grown after mixing equal volumes of protein solution (concentrated at 20 mg ml^−1^) and mother liquor consisting of 100 mm sodium cacodylate, pH 6.5, 20% w/v PEG 3350. The crystals appeared within 4–7 days reaching their final size in 2 weeks at 16 °C. Crystals were soaked in a cryoprotectant solution containing the mother liquor and 20% v/v ethylene glycol for 5–10 s prior their exposure to the beam. These crystals were tested on beamline X06DA at the Swiss Light Source (Villigen, Switzerland), and a single wavelength dataset was collected to 1.48 Å resolution. Images were indexed, integrated, and scaled using XDS (27) in the space group that was determined with POINTLESS (28) using unmerged data (Table 2). The structure was solved with molecular replacement in PHASER (29) using as a model the structure of BC0361 (Protein Data Bank code 4HD5) (23) and was subsequently refined in PHENIX (30). The asymmetric unit contained two molecules of BA0330, which superimpose almost perfectly with a root mean square deviation of 0.234 Å for the main chain atoms. Regular inspection of the electron density maps 2*F_o_* − *F_c_* and *F_o_* − *F_c_* and refitting of the model, where necessary, were performed with COOT (31). The refinement statistics for the converged final model are given in Table 2. Figures were generated using the PyMOL Molecular Graphics System, Schrödinger LLC.

**TABLE 2:** **Crystallization conditions, data collection, and refinement statistics** Ligands include all atoms, excluding protein and solvent atoms. *R*_meas_ = Σ*_hkl_* {*N*(*hkl*)/(*N*(*hkl*) − 1)}^1/2^ Σ*_i_*|*I_i_*(*hkl*) − 〈Ι(*hkl*)〉|/Σ*_hkl_*Σ*_i_I_i_*(*hkl*), where *N*(*hkl*) is the multiplicity, and *I_i_* is the intensity for the *i*th measurement of an equivalent reflection with indices *h*, *k,* and *l. r* = Σ*_hkl_*‖*F*_obs_| − |*F*_calc_‖/Σ*_hkl_*|*F*_obs_|, where *F*_obs_ and *F*_calc_ are the observed and calculated structure factors. *R*_free_ is calculated analogously for the test set reflections. ASU is asymmetric unit; r.m.s.d. is root mean square deviation. Values in parentheses correspond to the highest resolution shells.

**Data collection**	
Space group	C2
Cell dimensions	
*a, b, c* (Å)	108.96, 68.76, 132.02
α, β, γ (^ο^)	90.00, 96.86, 90.00
Wavelength (Å)	1.000
Resolution (Å)	48.00-1.48 (1.53-1.48)
Protein molecules/ASU	2
Unique reflections	155,203
*R*_meas_	0.062 (0.670)
〈*I*〉/〈σ*I*〉	17.8 (2.0)
Completeness (%)	96.3 (93.2)
Redundancy	4.6 (4.2)

**Refinement**	
Reflections, work/test set	147,443/7760
*R*_work_/*R*_free_	0.157/0.175
No. of atoms	
Protein/ligands/water	5140/34/943
Average B factor (Å^2^)	
Protein/ligand/water	23.7/26.6/33.3
Bond lengths r.m.s.d. (Å)	0.010
Bond angles r.m.s.d. (°)	1.024
Most favored	97%
Outliers	0.5%
Protein Data Bank code	4V33
Crystallization condition	100 mm sodium cacodylate, pH 6.5, 20% w/v PEG 3350
Cryo-protection solution	25% ethylene glycol

##### Preparation of Radiolabeled Substrate

Labeling of glycol chitin was performed using [^3^H]acetic anhydride according to Araki *et al.* (32).

##### Enzyme Assays

Enzyme assays were performed at a wide pH range and in the presence/absence of the divalent cations Co^2+^, Zn^2+^, Mn^2+^, Mg^2+^, Ni^2+^, Cu^2+^, and Cd^2+^. We have employed two different assays for determining PDA activity, namely a radiometric assay (32) and an assay based on fluorogenic labeling with fluorescamine (16).

##### Construction of B. anthracis Δba0330, Δba0331, and Δba0330/0331 Mutants, Complemented Strains, and GFP Fusions

DNA fragments containing the sequence upstream and downstream of *ba0330, ba0331,* and *ba0330/0331* were generated by PCR using the appropriate oligonucleotides (supplemental Table S1). Each fragment was cloned into pGEM vector. The constructs were then digested with SmaI/PstI to ligate the upstream and downstream fragments of each gene in the same plasmid. The proper cassettes that give resistance to spectinomycin (Spc) (33) from pSPCH+1, pSPCH+2, and pSPCH+3 were incorporated in-frame between the upstream and downstream fragments of each gene (34). After digestion, the whole construction (upstream fragment, Spc cassette, and downstream fragment) was ligated into the shuttle vector pUTE538 (35), and the construct was passaged through *E. coli* GM48 (*dam*^−^) to obtain nonmethylated plasmid DNA for electroporation into *B. anthracis*. To isolate a double-crossover recombinant Spc-resistant strain, transformants were grown in BHI medium with Spc for 2 days, diluted 1:1,000 every 12 h, and then shifted to BHI medium without antibiotic to facilitate clearance of autonomous plasmids. The culture was diluted 1:1,000 in fresh medium every 12 h for several days and then plated onto BHI agar with Spc. Colonies were patch-plated to score clones for Spc resistance and erythromycin sensitivity. Δ*ba0330*, Δ*ba0331,* and Δ*ba0330*/Δ*ba0331* in *B. anthracis* were also confirmed by PCR amplification.

Complementation studies were carried out as follows. The genes were amplified from genomic DNA of *B. anthracis* UM23C1-2 using DNA polymerase chain reaction. Primers were synthesized to incorporate a KpnI site at the start and a BglII site at the end of *ba0330* and *ba0331* genes. The amplified genes were purified, digested with the corresponding enzymes, and ligated into pWH1520 vector (36), placing the two genes under a xylose-inducible promoter. The constructs were passaged through *E. coli* GM48 (*dam*^−^) to obtain nonmethylated plasmid DNA and electroporated into *B. anthracis*. BHI medium was inoculated from overnight cultures to an *A*_600_ of 0.05 and incubated at 37 °C on a shaker incubator to an *A*_600_ of 0.6, where induction was achieved with 0.1% xylose (for expression from the xylose-inducible promoter).

To construct strains expressing GFP translational fusions, the *gfp-mut1* gene was amplified from pNF8 (37) with specific primers, digested with SphI and BglII, and ligated into the xylose-inducible plasmid pWH1520. Then each gene (lacking the stop codon) was amplified from *B. anthracis* UM23C1-2 chromosomal DNA with the appropriate primers to incorporate at the C terminus the polylinker GPGP. The amplicon was digested with KpnI and SphI and ligated in-frame to the 5′ end of *gfp-mut1. B. anthracis* cells were then transformed with the resulting plasmids via electroporation, after initially being passaged through *E. coli* GM48 (*dam*^−^) to obtain nonmethylated plasmid DNA. 10 ml of BHI medium were inoculated from overnight cultures to an *A*_600_ of 0.05 and incubated at 37 °C with shaking to an *A*_600_ of 0.6, where induction was achieved with 0.1% xylose (final concentration).

##### Peptidoglycan and Neutral Polysaccharide Purification

PG from parental *B. anthracis* UM23C1-2 and mutants was prepared from exponentially and stationary phase growing bacteria and purified as described previously (38). Muropeptides from the native PG were generated using the muramidase cellosyl, separated by HPLC, purified, and analyzed by mass spectrometry as described previously (38). Neutral polysaccharide was extracted and purified from cell walls as described by Ekwunife *et al.* (39).

##### Autolysis Assay

Autolysis assay was performed according to Balomenou *et al.* (15).

##### Fluorescence Microscopy of Vegetative Cells

*B. anthracis* cultures of the parental and mutant strains were inoculated from fresh overnight plates to an initial *A*_600_ of 0.1 and grown to stationary phase as liquid cultures in SPY medium. Cells were examined by fluorescence microscopy, and the images were obtained without fixation on an inverted epifluorescence microscope Nikon E800.

##### Western Blotting Analysis

Bacterial cell lysates during the time points at which GFP fluorescence signal was obtained were separated by SDS-PAGE, blotted, and probed with the following antibodies: polyclonal rabbit anti-GFP primary antibody diluted 1:5,000 and polyclonal goat anti-rabbit IgG horseradish peroxidase secondary antibody diluted 1:50,000.

##### Transmission Electron Microscopy

For transmission electron microscopy (TEM), cells were fixed with 2.5% glutaraldehyde in 0.1 m cacodylate buffer, pH 7, and post-fixed in 1% osmium tetroxide. Samples were pelleted and embedded in low melting point 2% agar, transferred in 0.5% uranyl acetate, and then dehydrated and embedded in epoxy resin. Sections were observed in a JEOL, JEM 2100 transmission electron microscope, operated at 80 kV.

##### Scanning Electron Microscopy

For scanning electron microscopy (SEM), samples were fixed in 2% glutaraldehyde, 2% paraformaldehyde in 0.1 m sodium cacodylate buffer, pH 7.4, dehydrated, and then dried and mounted prior to sputter coating with 20 nm thickness gold/palladium Sputter Coater. Samples were examined using a JEOL JSM-6390LV scanning electron microscope, operating at 15–20 kV.

##### In Vitro Determination of Lysozyme Resistance

To test the sensitivity of the mutants in the presence of exogenously added lysozyme, overnight cultures of the parental and mutant strains were diluted to an *A*_600_ of 0.1 in 1 liter of fresh SPY medium. The cultures were incubated at 37 °C until an *A*_600_ of 1.0. Then each culture was divided in two equal parts of 500 ml, and 10 μg ml^−1^ hen egg lysozyme was added at one of the two subcultures. The growth of both treated and untreated subcultures was monitored.

##### Salt Stress Adaptation

To study the effects of exposure to mild (2.5 and 3.5%) and severe (4.5%) salt stress on the growth of *B. anthracis* UM23C1-2 and mutants strains, stationary phase cultures were diluted 1:100 (v/v) in flasks containing 50 ml of fresh BHI broth and incubated at 37 °C with shaking at 200 rpm. When an absorbance at *A*_600_ of 0.6 was reached, BHI broth supplemented with 2.5, 3.5, and 4.5% (w/v) NaCl (final supplementary concentrations) was inoculated at a starting *A*_600_ of 0.01. The cultures were incubated further at 37 °C with shaking at 200 rpm, and growth was monitored for 9 h. To study the effects of exposure of severe salt stress on the growth of several mutant strains of *B. anthracis*, stationary phase cultures were diluted 1:100 (v/v) in flasks containing 50 ml of fresh BHI broth and incubated at 37 °C with shaking at 200 rpm. When *A*_600_ of 0.6 was reached, strains were plated on BHI solid medium containing 4.5% (w/v) NaCl (final supplementary concentration) and further incubated at 37 °C.

##### PG Binding Assay

Purified PG (100 μg) from *B. anthracis* was incubated with purified BA0330 and BA0331 (30 μg) in 20 mm Tris-HCl, pH 8.0, in a final volume of 90 μl for 30 min at 4 °C with agitation. The suspension was centrifuged for 10 min at 15,000 × *g*, and the supernatant (soluble fraction) was kept for further analysis. The pellet was washed twice with 250 μl of buffer and suspended in 90 μl of buffer (insoluble fraction). Unbound proteins in the soluble fractions and bound proteins in the insoluble fractions were analyzed by SDS-PAGE.

##### Site-directed Mutagenesis of BA0330 and BA0331

Mutants were constructed using a two-step/four-primer overlap extension PCR method (40). The amplified products were subcloned into the pWH1520 vector, and mutant cells were transformed and examined by TEM.

## Results

### 

#### 

##### BA0330 and BA0331 Are Predicted Lipoproteins

**FIGURE 1.**
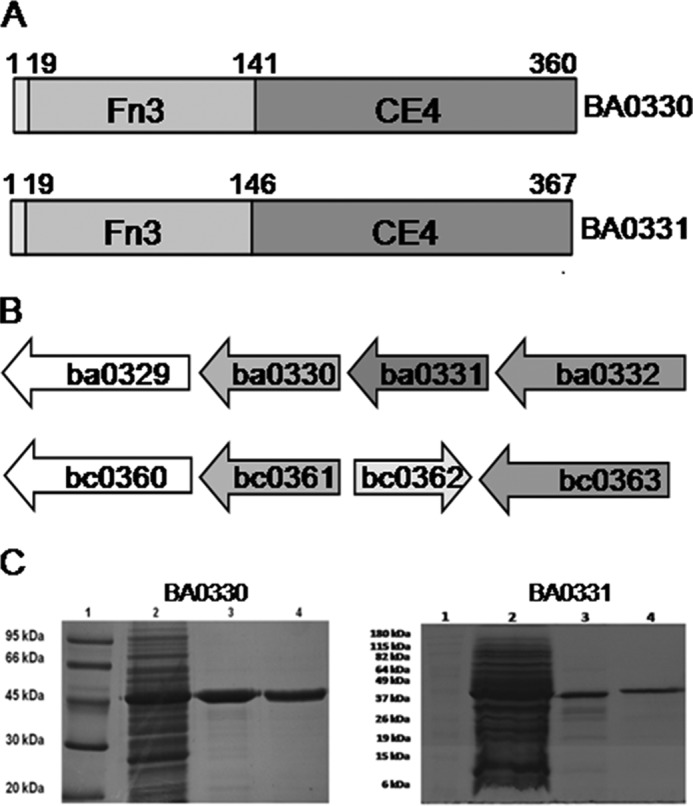
*A,* schematic representation of BA0330 and BA0331 domains. *B,* gene organization of putative PDAs *ba0330/ba0331* and *bc0361* in the genomes of *B. anthracis* UM23C1-2 and *B. cereus* ATCC 14579. These genes are flanked by the putative aminopeptidase genes *ba0329* and *ba0360* and NupC-like nucleoside transporters *ba0332* and *bc0363. Arrows* indicate open reading frames. Homologous genes are indicated with the same shading. *C,* SDS-PAGE of the purified putative PDAs BA0330 and BA0331. For BA0330: *lane 1*, molecular weight markers; *lane 2*, crude extract; *lane 3*, SP-Sepharose eluate; *lane 4*, gel filtration eluate. For BA0331: *lane 1*, molecular weight markers; *lane 2*, crude extract; *lane 3*, Source Q eluate; *lane 4*, gel filtration eluate. Samples were electrophoresed on a 12% polyacrylamide gel under denaturing and reducing conditions. Protein bands were visualized by staining with Coomassie Brilliant Blue R.

The genome of *B. anthracis* Ames sequence database (GenBank^TM^ accession number AE016879) reveals 11 coding sequences for putative PDAs of family CE4. Ten of them exhibit more than 90% sequence identity to their homologues from *B. cereus sensu stricto* (Table 3). For two of them, BA0330 and BA0331, the programs SignalP and TatP predicted a signal peptide for targeting to the Sec translocation pathway. The LocateP program predicted them to be N-terminally anchored membrane proteins with a characteristic lipobox consensus sequence (LVI)(ASTVI)(GAS)(C), which is the hallmark for lipid modification of proteins in bacteria. In BA0330 and BA0331 Cys^19^ is the lipid-modified cysteine residue (Fig. 1*A*). *ba0330* and *ba0331* reside in an operon with *ba0329*, a putative aminopeptidase encoding gene (Fig. 1*B*).

**TABLE 3:** **The 11 coding sequences from *B. cereus* and *B. anthracis*** Values in parentheses refer to the number of amino acids in the respective open reading frame. Possible function has been assigned to these enzymes by ERGO-light data base. MurNAc, *N*-acetylmuramic acid.

*B. cereus* ATCC 14579	*B. anthracis* st. Ames	Possible function	Identity	Similarity
NP_831730 (275) (BC1960)	NP_844369 (275) (BA1961)	Peptidoglycan GlcNAc deacetylase	94	97
NP_833348 (213) (BC3618)	NP_845942 (213) (BA3679)	Peptidoglycan GlcNAc deacetylase	97	100
NP_832677 (275) (BC2929)	NP_845280 (275) (BA2944)	Peptidoglycan GlcNAc deacetylase	94	97
NP_834868 (245) (BC5204)	NP_847604 (245) (BA5436)	Peptidoglycan GlcNAc deacetylase	93	96
NP_831744 (273) (BC1974)	NP_844383 (273) (BA1977)	Peptidoglycan GlcNAc deacetylase	98	99
NP_830306 (260) (BC0467)	NP_842967 (273) (BA0424)	Peptidoglycan MurNAc deacetylase	98	99
NP_830050 (254) (BC0171)	NP_842717 (254) (BA0150)	Chitooligosaccharide deacetylase	95	99
NP_831543 (234) (BC1768)	NP_844255 (234) (BA1836)	Chitooligosaccharide deacetylase	92	96
NP_833526 (299) (BC3804)	NP_846187 (299) (BA3943)	Chitooligosaccharide deacetylase	95	97
NP_830200 (360) (BC0361)	NP_842877 (360) (BA0330)	PDA	91	94
NP_830200 (360) (BC0361)	NP_842878 (367) (BA0331)	PDA	53	69

##### BA0330 and BA0331 Lack Deacetylase Activity against Common Substrates

*ba0330* and *ba0331* were expressed in *E. coli,* and recombinant proteins were purified to near homogeneity (Fig. 1*C*). Both recombinant proteins appear to be monomers as revealed by gel filtration chromatography (data not shown). To examine their substrate specificity, several commonly used deacetylase substrates were tested in enzyme assays. BA0330 and BA0331 were not active against radiolabeled glycol chitin, *N*-acetyl chitooligosaccharides, the synthetic muropeptide *N*-acetyl-d-glucosaminyl-(β-1,4)-*N*-acetylmuramyl-l-alanyl-d-isoglutamine, and *p*-nitrophenyl acetate when tested at a wide pH range and in the presence or absence of the divalent cations Co^2+^, Zn^2+^, Mn^2+^, Mg^2+^, Ni^2+^, Cu^2+^, and Cd^2+^. Both proteins were also inactive against the same substrates when they were purified from *B. anthracis* (data not shown), excluding that the lack of activity was due to their production in *E. coli* as recombinant proteins.

##### Structure of BA0330 Reveals Unique Features of the Active Site

**FIGURE 2.**
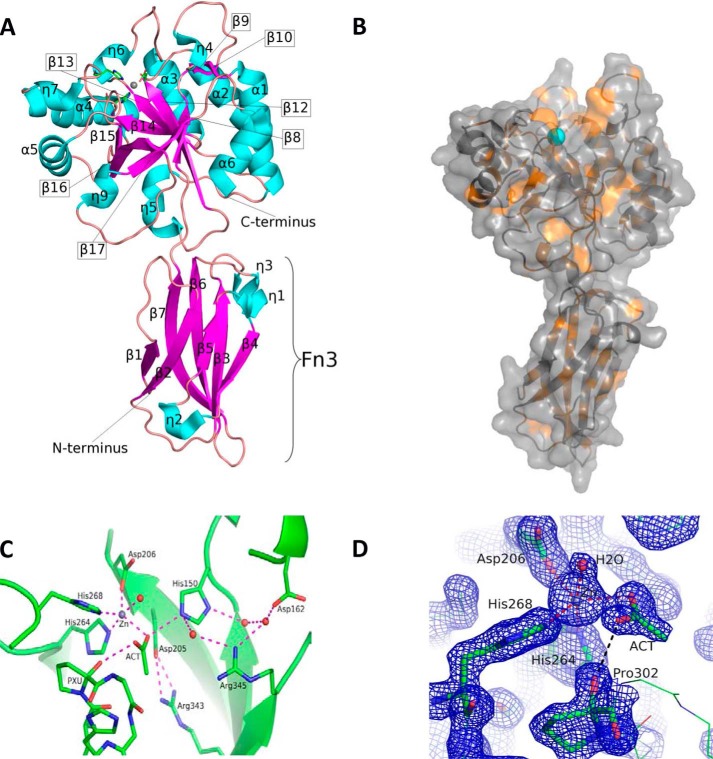
*A,* overall structure of BA0330. Helices are represented with *cyan ribbons* and β-strands with *magenta* and loops as *pink strings*. Zn^2+^ is shown as a *gray sphere* with the coordinating amino acid residues (Asp^206^, His^264^, and His^268^) in *stick* representation. *B,* surface representation of the BA0330 molecule showing the CE4 domain (*top*) and the Fn3 domain (*bottom*). The metal-containing binding cavity is located at the top of the molecule with the zinc atom (shown in *cyan*) exposed at the bottom of the cavity. Hydrophobic residue side chains (Leu, Ile, Val, Ala, Gly, Phe, Trp, and Met) are shown in *orange* and the rest in *gray. C,* putative binding site of BA0330 containing the zinc coordination residues (Asp^206^, His^264^, and His^268^), the acetate ion, and a water molecule shown as a *red sphere*. Additionally, the catalytic residues Asp^205^, Arg^343^, His^150^, Asp^162^, and Arg^345^ are shown, and the whole network of interactions, either direct or through water molecules, is presented in *dashed magenta lines. D,* BA0330 zinc-containing site in detail, shown with the 2*F_o_* − *F_c_* electron density map contoured at 2σ level (*blue*). The zinc coordinating residues are shown in *stick* representation.

We next determined the crystal structure of BA0330 at 1.48 Å (Fig. 2*A*), which revealed two domains. In addition to the CE4-type esterase domain, there is an N-terminal fibronectin type 3 (Fn3)-like domain (residues 45–141) consisting of a two-layered (4 + 3) β-sandwich (Fig. 2*A*). Apart from the closely related BC0361 and BA0331 no other CE4 esterase studied so far has an Fn3-like domain. Part of the expressed protein (residues 24–44 of the N terminus) could not be determined in the electron density maps, and therefore the corresponding region was not built in the model. The CE4 domain resembles that of other deacetylases, containing a well formed groove with the zinc atom located at the bottom (Fig. 2*B*).

**FIGURE 3.**
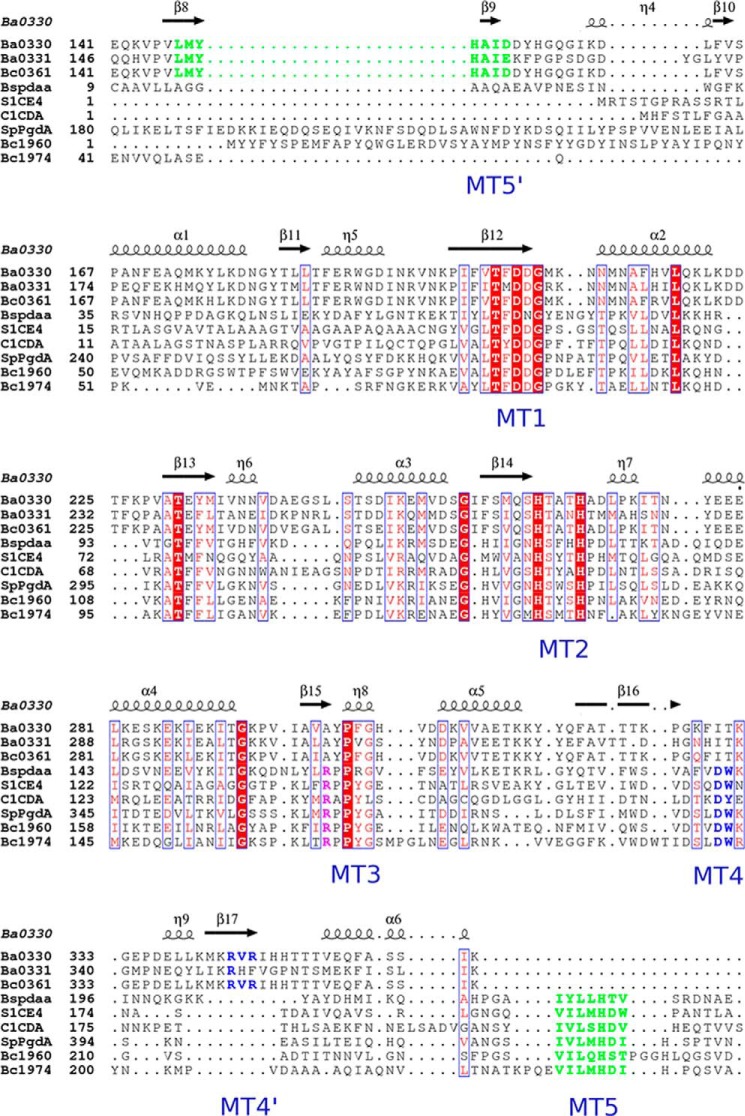
**Sequence alignment of the NodB domains of the BA0330 and BA0331 proteins and representative members of the CE4 family, including the putative PDA BC0361, the chitin deacetylase ClCDA, the acetylxylan esterase SlCE4, and the peptidoglycan deacetylases SpPdgA, BsPdaA, BC1960, and BC1974.** The secondary structure elements of the BA0330 structure are shown at the *top* of the alignment, and the CE4 active-site motifs (MT1 to MT5) are shown at the *bottom*. The alignment was performed with T-coffee (64) and plotted with the ESPRIPT (65). Strictly conserved residues are colored *white* on a *red background,* and similar residues are *red* and *boxed*. The residues participating at the MT4 and MT5 motifs are shown in *blue* and *green*, respectively. Structural alignment of the proteins that contain an Fn3-like domain demonstrates a charge reversal in the MT4 region labeled as *MT4′*. Similarly, they present a shift of the MT5 region, which is located close to the N terminus of the NodB domain (labeled as *MT5′*).

**FIGURE 4.**
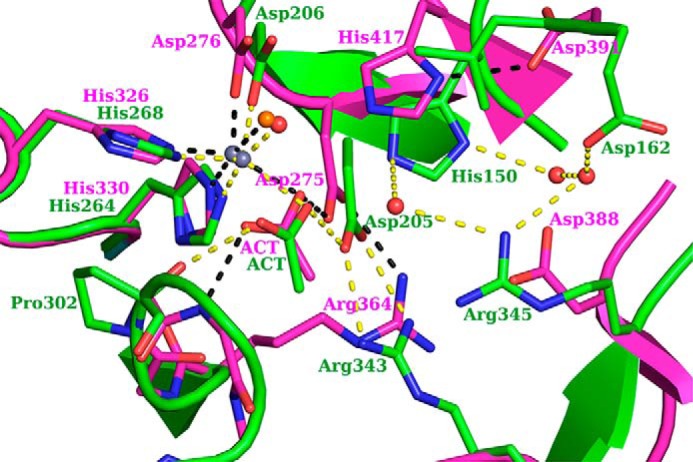
**Superposition of the binding sites of SpPgdA in *magenta* (Protein Data Bank code 2C1G) and BA0330 in *green*.** The residues involved in interactions are shown in *stick* representation, BA0330 water molecules as *red spheres*, SpPgdA water molecule as *orange sphere,* and the interactions in *yellow* and *black dashed lines* for BA0330 and SpPgdA structures, respectively. BA0330 residues are labeled in *green*, and the SpPgdA ones are in *magenta*.

BA0330 and BA0331 retain the classical Asp-His-His arrangement within the sequence alignment, suggesting that they could bind a metal cation within their binding site, and they contain most of the catalytic and zinc-binding residues conserved in five catalytic motifs of enzymatically and structurally characterized CE4 esterases, including chitin deacetylase ClCDA, PG deacetylases SpPgdA, BsPdaA, BC1960, and BC1974, putative PDA BC0361, and acetylxylan esterase SlCE4 (Fig. 3). However, arrangements and alterations in both proteins occurred in motifs 4 and 5. Specifically, motif 5 is located close to the N terminus of the NodB domain, and motif 4 is shifted toward the C terminus. Additionally, in both proteins motif 4 is electrostatically altered, as the conserved aspartic acid present in the other members of the CE4 family (Asp^388^ in SpPgdA), is replaced by an arginine (Arg^345^ in BA0330). The electron density at the binding site was attributed to the zinc ion (Fig. 2*D*), which is coordinated in a trigonal bipyramidal manner by two histidines, one aspartic acid, a water molecule, and an acetate molecule, probably present as a contaminant to the PEG solutions used in the crystallization buffer (16). The acetate ion was well ordered (present up to 6σ in the *F_o_* − *F_c_* map), with an average B-factor of 27 Å^2^ close to the corresponding value for the zinc atom (Fig. 2*C*). The acetate molecule interacts extensively with residues of the binding site. One of its oxygens interacts with the invariant Asp^205^, which is tethered with Arg^343^ (present only in Fn3 domain-containing deacetylases), whose guanidinium group overlaps spatially with the corresponding group of SpPgdA-Arg^364^ (Fig. 4). Furthermore, the same oxygen atom seems to interact with the structurally conserved His^150^, which, unlike other deacetylases, does not interact directly with closely placed amino acid residues. The other acetate oxygen accepts a hydrogen bond at 2.9 Å (Fig. 2*D*) from the hydroxyl group of the modified proline (Pro^302^), which was modeled as an α-hydroxy-l-proline located at a distance of 3.6 Å from the metal ion. The terminal methyl group of the acetate occupies a small hydrophobic patch generated by the methyl group of Thr^324^ and Met^148^. The aspartic acid in motif 1 (BA0330 numbering Asp^205^) acts as a catalytic base by activating the nucleophilic H_2_O, and the histidine (BA0330 numbering His^150^) acts as the catalytic acid (Fig. 2*C*). The interaction of the catalytic base (Asp^205^) with an arginine residue (BA0330 numbering Arg^343^) is essential for catalysis.

Therefore, the catalytic site differs from that typically found in PDAs in the following points. (i) The Arg that interacts with the catalytic Asp is usually found at the start of motif 3. Here, this Arg^343^ is provided from the last β-strand (β17, Fig. 3). (ii) The catalytic histidine (His^150^), which is usually within motif 5, is located at a nonconserved sequence position in BA0330. (iii) BA0330 lacks the aspartic acid, which is usually provided by motif 4, and it is believed to be responsible for tuning the p*K_a_* value of the catalytic histidine (His^150^). Instead, the structure of BA0330 reveals an arginine residue (Arg^345^) in close proximity to His^150^ and a possible interaction between them through a water molecule. In addition, Asp^162^ may interact with the catalytic His^150^ through two reactive water molecules (Fig. 2*C*).

##### Phenotypic Analysis Reveals Roles of ba0330 and ba0331 in Autolysis and Cell Shape

To elucidate the biological roles of BA0330 and BA0331, the single mutants Δ*ba0330* and Δ*ba0331* and the double mutant Δ*ba0330*Δ*ba0331* were constructed in *B. anthracis* UM23C1-2 (pXO1- and pXO2-). All mutant cells were able to grow in various liquid media (BHI broth and SPY medium (41)), indicating that the genes were not required for *B. anthracis* viability and growth (data not shown).

**FIGURE 5.**
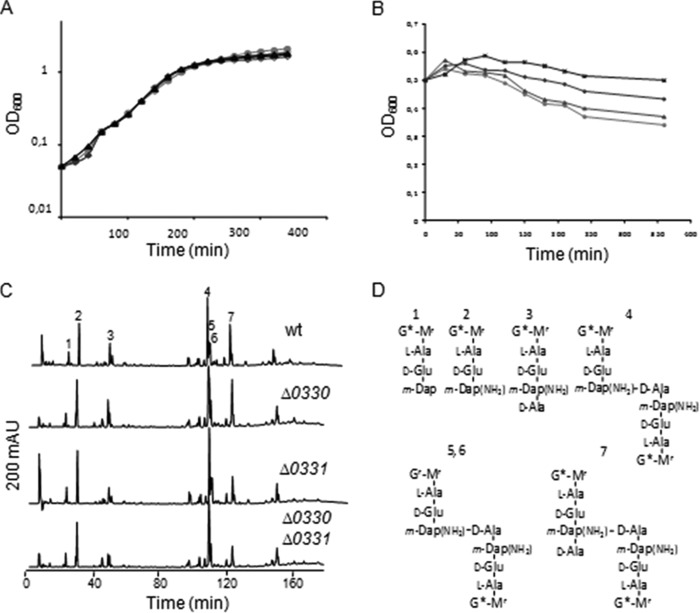
**Lysozyme sensitivity, autolysis rate, and muropeptide analysis of parental and mutant strains.** *A,* effect of lysozyme on UM23C1-2 (○), Δ*ba0330* (♢), and Δ*ba0331* (▵) mutant strains. Strains were grown in SPY liquid broth at 37 °C. *Closed symbols* indicate the corresponding strains treated with 10 μg ml^−1^ hen egg lysozyme. None of the mutant strains were affected by the addition of lysozyme. *B,* autolysis rate of UM23C1-2 and mutant strains. Autolysis was induced by the addition of 10 mm sodium azide to cultures of *B. anthracis* UM23C1-2 parental strain (●) and Δ*ba0330* (♦), Δ*ba0331* (▴), and Δ*ba0330*Δ*ba0331* (■) derivative strains. Cell lysis was monitored by loss of absorbance at 600 nm. Δ*ba0330*Δ*ba0331* strain was not affected by autolysis; Δ*ba0330* strain was affected to a lesser degree compared with the wild type; and Δ*ba0331* strain exhibited a similar pattern to the wild-type strain. *C,* HPLC analysis of muropeptide composition of PG from vegetative cells of UM23C1-2, Δ*ba0330*, Δ*ba0331,* and Δ*ba0330/0331*. *Peaks 1–7* were analyzed by mass spectrometry (data not shown). *mAU* indicates the absorbance where muropeptides are detected in the chromatograph. *D,* proposed structures of major muropeptides *1–7* detected in *C* peak identification. Deacetylated monosaccharides are indicated with the following: *, *G*, *N*-acetylglucosamine; G*, glucosamine; *M_r_*, *N*-acetylmuramitol; *m-Dap*(*NH*_2_), amidated *meso*-diaminopimelic acid.

Many bacterial PG deacetylases described so far contribute to lysozyme resistance (10, 42–44). However, Δ*ba0330* or Δ*ba0331* mutant cells grown in SPY medium did not display an altered sensitivity to lysozyme compared with the wild type in the exponential or stationary growth phases (Fig. 5*A*).

We next tested the autolytic activity of parental *B. anthracis* UM23C1-2 strain and the putative PDA mutants by addition of NaN_3_, a known inducer of autolysis in growing cells (45). Δ*ba0330* and to a lesser extent Δ*ba0331* mutant strains showed decreased autolysis under these conditions compared with that of the parental strain, whereas Δ*ba0330*Δ*ba0331* did not lyse (Fig. 5*B*), indicating that both proteins affect the function of one or more autolysins.

Because PG is a major polysaccharide of *B. anthracis*, we determined the muropeptide composition of the PG of each mutant. The PG composition from the exponential phase cells of Δ*ba0330*, Δ*ba0331,* and Δ*ba0330*Δ*ba0331* mutants and of the parental strain UM23C1-2 were analyzed by HPLC (Fig. 5*C*), and the main muropeptides were identified by mass spectrometry (Fig. 5*D*). The overall muropeptide profiles derived from all mutant strains were similar to that of the parental strain, with the exception of the abundance of tetrapeptides and tripeptides, which varied between wild-type and Δ*ba0331* and Δ*ba0330*Δ*ba0331* mutant strains (Fig. 5*C*). Importantly, there was no significant difference in the abundance of deacetylated muropeptides. Isolated neutral polysaccharide from Δ*ba0330*, Δ*ba0331,* and Δ*ba0330*Δ*ba0331* mutant strains displayed identical chromatograms (*A*_206_) to that of the wild-type strain (data not shown). These results indicate that BA0330 and BA0331 do not function to significantly change the level of *N*-acetylation of the PG and the neutral polysaccharide.

**FIGURE 6.**
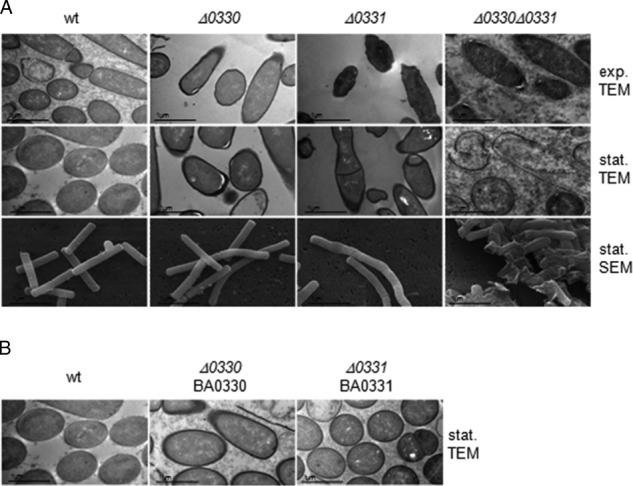
**Phenotype analysis of parental and mutant strains.** *A,* transmission electron micrographs of UM23C1-2 and Δ*ba0330*, Δ*ba0331*, Δ*ba0330/0331* mutant cells during vegetative and stationary growth. Scanning electron micrographs of UM23C1-2 and Δ*ba0330*, Δ*ba0331*, Δ*ba0330/0331* mutant cells during stationary growth. Δ*ba0330* strain exhibited sites of detachments of PG from the cell membrane; Δ*ba0331* an atypical cell shape and Δ*ba0330/0331* exhibited a virtually complete detachment of the membrane from the cell wall and formation of aggregates during stationary growth phase. *Exp.* indicates exponential growth, and *Stat* indicates stationary growth. *B,* transmission electron micrographs of UM23C1-2 and Δ*ba0330*, Δ*ba0331,* mutant strains complemented with BA0330 and BA0331, respectively, during stationary growth. Both Δ*ba0330* and Δ*ba0331* recovered wild-type (*wt*) phenotype after *trans* complementation with BA0330 and BA0331, respectively.

We next used electron microscopy to further investigate possible cell wall alterations in the mutant strains (Fig. 6*A*). Exponentially and late stationary phase growth cells were fixed and imaged by TEM and SEM. Although wild-type cells showed the typical appearance for Gram-positive bacilli, the mutant cells exhibited different phenotypes. In Δ*ba0330* cells, we observed a partial detachment of the membrane from the cell wall, presumably due to a weakened interaction between the two layers. In contrast, Δ*ba0331* cells had a normal PG-membrane connection but showed a distorted cell shape. The morphological changes were best seen by SEM showing variable cell diameter in stationary Δ*ba0331* cells and extensive clumping and lysis of Δ*ba0330*Δ*ba0331* cells. These maintained the partial cell wall detachment from the membrane during vegetative growth and showed extensive lysis during stationary growth, indicating that both proteins together are needed to maintain cell shape and integrity. The complemented strains fully recovered the wild-type phenotype when imaged by TEM (Fig. 6*B*). We have observed the same phenotypes in three independent experiments, thus excluding the possibility of EM artifacts.

##### Cells Lacking ba0330 and ba0331 Are More Sensitive upon Salt Upshift

**FIGURE 7.**
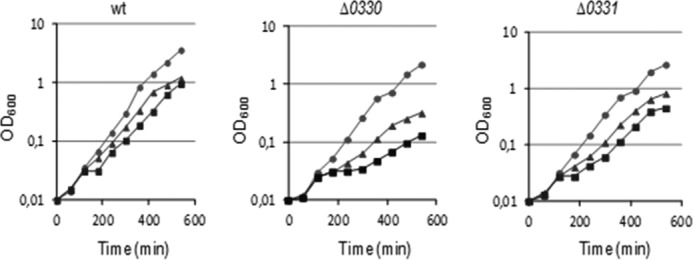
**Effect of different NaCl concentrations on growth kinetics.** Exponentially growing cells of parental strain and mutants were challenged with 2.5% NaCl (●), 3.5% NaCl (▴), and 4.5% NaCl (■), and the changes in growth kinetics were monitored by changes in absorbance at 600 nm. Δ*ba0330* showed greater sensitivity at increasing concentrations of NaCl, whereasΔ*ba0331* was less affected.

Previous studies in Gram-positive bacteria *B. subtilis* and *Lactobacillus casei* revealed that high salt (NaCl) concentration reduced growth and resulted in detachment of cell wall from the cytoplasmic membrane (46, 47). We therefore tested the growth of our mutants in the presence of increasing NaCl concentrations. In contrast to the wild-type strain, both mutant strains grew slower at high NaCl concentrations (Fig. 7), a phenotype that was much more pronounced in Δ*ba0330* for concentrations higher than 3.5% NaCl. Moreover, the cell clumping and formation of filamentous cells forming visible aggregates was observed at a 3.5% NaCl concentration and enhanced at higher salt conditions. These effects were enhanced in Δ*ba0330*Δ*ba0331* cells even under mild salt stress conditions (2.5%), preventing reliable growth monitoring for this strain. Therefore, we conclude that BA0330, and to a less extent BA0331, is important for adaptation of *B. anthracis* to grow at high salt concentrations.

##### BA0330 and BA0331 Interact with Peptidoglycan

**FIGURE 8.**
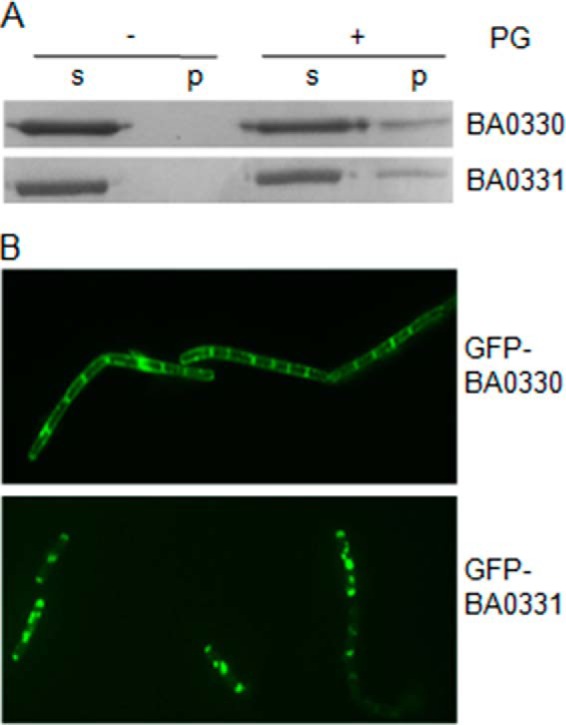
*A,* BA0330 and BA0331 interact with PG. Purified BA0330 and BA0331 proteins were incubated with or without PG followed by sedimentation of the PG by ultracentrifugation. PG was washed and sedimented again before proteins were separated by SDS-PAGE and visualized by Coomassie staining. A fraction of each protein was pulled down with PG. *s*, supernatant after centrifugation; *p*, resuspended pellet. *B,* localization of GFP-BA0330 and GFP-BA0331. Fusion proteins were constructed using the *B. anthracis* UM23C1-2 parental strain and were functional. GFP-BA0330 localizes at the cell periphery and is enhanced at the septa. GFP-BA0331 localizes at distinct spots at periphery and exhibits low fluorescence at septa. *Scale bars*, 5 μm.

A pulldown assay was used to investigate whether the two proteins bind to insoluble PG. Both BA0330 and BA0331 were found to interact with isolated PG from each of the corresponding mutant strains, as the proteins were detected in the pellet fraction after the pulldown experiment (Fig. 8*A*). These results suggest that the two lipoproteins, which are attached via their lipid moiety to the cell membrane, span to the PG layer and interact with the PG layer.

##### GFP Fusions of BA0330 and BA0331 Localize to Lateral Wall and Distinct Foci, Respectively

**FIGURE 9.**
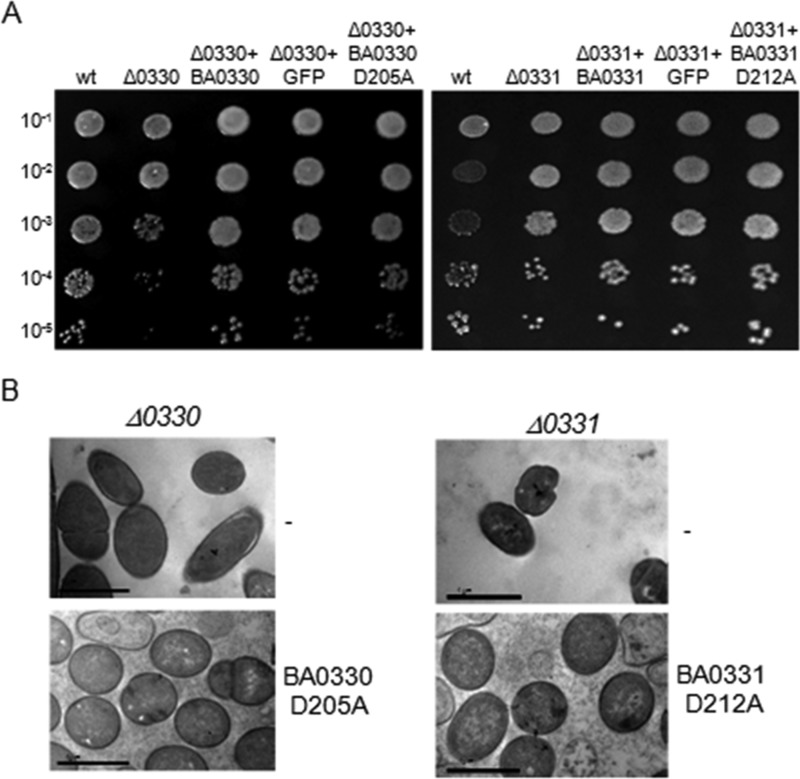
*A,* growth of Δ*ba0330* and Δ*ba0331* mutant strains complemented with BA0330 and BA0331, GFP-BA0330 and GFP-BA0331, and BA0330 (D205A) and BA0331 (D212A), respectively, under high NaCl concentration (4,5%). *B. anthracis* UM23C1-2 was used as a control. Whereas increased salt concentration inhibited growth of Δ*ba0330* and Δ*ba0331* mutant strains compared with the wild-type (*wt*) strain, all complemented strains recovered the wild-type phenotype. *Numbers* on the *left axis* indicate dilution factors of the cultures. *B,* site-directed mutagenesis of a predicted key enzymatic residue of BA0330 and BA0331. BA0330 and BA0331 with a point mutation in a key catalytic residue were expressed in Δ*ba0330* and Δ*ba0331* cells, respectively. Cells were grown until late stationary phase, and samples for TEM observation were prepared. Δ*ba0330* exhibited characteristic detachments, whereas the same strain complemented with BA0330 (D205A) recovered the phenotype of the parental strain. Δ*ba0331* cells exhibited irregular cell shape, and the same strain complemented with BA0331 (D212A) recovered the typical bacilli shape.

To gain more insights into the physiological roles of the two putative deacetylases, we determined the subcellular localization of the C-terminal GFP-fused proteins by fluorescence microscopy in UM23C1-2 cells (Fig. 8*B*). Fluorescent signal was obtained for each deacetylase during the exponential phase of growth, and the expression of the GFP-fused proteins was confirmed by Western blot analysis (data not shown). BA0330-GFP localized at the lateral wall of the cell and was enhanced at the septum. BA0331-GFP displayed a different localization pattern, with fluorescence label distributed as distinct patches with lower fluorescence at the septa. Membrane localization was consistent with the prediction of the two putative PDAs as lipoproteins. The GFP tag did not affect the function of the two proteins, because mutant strains transformed with the respective GFP-fused proteins exhibited the same phenotype as wild type when grown under high salt concentrations (Fig. 9*A*).

##### Deacetylase Activity Is Not Required to Complement Mutant Phenotypes

To examine the importance of the putative deacetylase activity of the proteins *in vivo*, a point mutation was introduced in *ba0330* and *ba0331* to replace a predicted key catalytic aspartic acid residue with alanine generating BA0330 (D205A) and BA0331 (D212A), respectively. A shuttle vector expressing the mutated genes was introduced in the *B. anthracis* mutant strains, and the resulting strains were examined by TEM. Interestingly, the mutant cells expressing the point mutated proteins lost their distorted phenotypes and fully recovered the wild-type phenotype (Fig. 9*B*), indicating that the enzymatic activity of the two proteins is not important for the observed phenotype of the mutants. Similarly, when inactive proteins were expressed in Δ*ba0330* and Δ*ba0331* mutant strains in the presence of 4.5% NaCl, the wild-type phenotype was restored (Fig. 9*A*). Hence, cell wall stability and cell shape maintenance do not require enzymatically active proteins.

## Discussion

In this study, we employed gene knock-out and structural analysis as well as protein localization studies to elucidate the physiological role of two lipoproteins, the putative polysaccharide deacetylases BA0330 and BA0331, in *B. anthracis*.

### 

#### 

##### Are BA0330 and BA0331 Inactive Deacetylase Versions?

Surprisingly, in contrast to most other PDAs, BA0330 and BA0331 were not active on a wide range of different substrates such as glycol chitin, *N*-acetylchitooligomers (GlcNAc_2–6_), *N*-acetyl-d-glucosaminyl-(β-1,4)-*N*-acetylmuramyl-l-alanyl-d-isoglutamine, *p*-nitrophenyl acetate. Furthermore, inactivation of *ba0330*, *ba0331*, and *ba0330/ba0331* did not result in a measurable change in the levels of PG deacetylation, as shown by our analysis of the muropeptide composition (Fig. 5, *C* and *D*).

The crystal structure of BA0330 has a well formed groove on the esterase domain that is lined with some hydrophobic residues and oriented toward the active-site residues (Fig. 2*B*). Furthermore, BA0330 and its homologue BC0361 from *B. cereus* contain a Cα-modified proline at its active site, which was modeled as an α-hydroxy-l-proline (23). How this proline becomes modified and whether this modification has any function is currently not known.

BA0330 has the characteristic zinc binding (His, His, and Asp) and catalytic (Asp and His) motifs of PDAs (Fig. 2*C*). Interestingly, Arg^345^ is found in place of a conserved aspartic acid that is believed to be responsible for tuning the p*K_a_* value of the catalytic histidine (His^150^) (Fig. 4). Regulation of enzyme activity, altered substrate specificity, or metal preference of CE4 family enzymes may be effected via modifications of the five CE4 motifs. It has been reported previously that mutation D391N in SpPgdA completely inactivated the enzyme (16). A similar observation has been reported for PgaB from *E. coli* whereby a water molecule is found in place of this conserved aspartic acid (19), possibly explaining the reduced efficiency of the enzyme. By superimposing the active sites of SpPgdA and BA0330 (Fig. 4), it is apparent that the conserved His p*K_a_* modulator (Asp^391^ in SpPgdA) is replaced by a Leu in BA0330. However, the nearby Arg^345^ in BA0330 may act as a titratable catalytic group (His^150^) modulator.

BA0330 and BA0331 lacked deacetylase activity in our assays, and their absence did not change PG deacetylation or lysozyme resistance (Fig. 5*A*), but both did bind to purified PG (Fig. 8*A*). Therefore, we consider the possibility that the main functions of these proteins do not involve an enzymatic activity but rather they have a structural, cell wall-stabilizing role. That putative enzymes are inactive and may have other enzymatic functions is not unprecedented. For example, the *E. coli* PG protein EnvC, classified as a LytM-type endopeptidase, was initially thought to be a septum-splitting PG hydrolase (48), until later studies revealed that EnvC and its homologue NlpD are inactive due to mutations in key catalytic residues, and that their main role is to activate the septum-splitting amidases (49). However, we cannot strictly exclude that BA0330 and BA0331 have an enzymatic activity that is limited to a restricted cell surface area and is not detectable by our techniques. Alternatively, they have an as-yet-unidentified substrate in the *B. anthracis* cell wall distinct from the neutral polysaccharide of *B. anthracis* or the deacetylase-specific GlcNAc residues for subsequent anchoring of cell wall polymers as demonstrated previously for BA1961 and BA3679 (50).

##### Role of the Fn3 Domain

The crystal structures of both BA0330 and BA0331 (BA0330 in this study; BA0331,^4^ currently under structural optimization) revealed the presence of an N-terminal Fn3 domain, which is unique for this family, and a C-terminal catalytic domain (Fig. 1*A*). Together with BC0361 from *B. cereus,* these are the first putative PDAs characterized to date with an Fn3 domain. These are often found in bacterial extracellular carbohydrases such as chitinases, amylases, cellulases, etc., and it is believed that they participate in promotion of the hydrolysis of carbohydrate substrates by modifying their surfaces, although they can also play important functional roles by formation of protein-protein interfaces (51). Fn3 domains are present in various PG-hydrolyzing enzymes, such as d,d-carboxypeptidases PPB5 and PBP6 in *E. coli* (52, 53). It has been previously proposed that differences in the Fn3 domains might affect protein localization, enzymatic activity, or interactions with other components of the PG biosynthetic machinery (53) It is possible that different localization of BA0330 and BA0331 is due to different functions of their Fn3 domains. The Fn3 domains of both proteins might be involved in their interaction with PG. However, we cannot exclude the possibility that they act as spacers between the membrane-linked N terminus and the catalytic domain (54). Experiments to clarify the roles of the Fn3 modules in the two lipoproteins are ongoing.

##### Do BA0330 and BA0331 Regulate ld-Carboxypeptidases?

Muropeptide composition analysis (Fig. 5, *C* and *D*) revealed that the Δ*ba0331* and Δ*ba0330/*Δ*ba0331* mutants exhibited reduced amounts of tetrapeptides with a concomitant increase in tripeptide content as compared with wild-type and the Δ*ba0330* mutant. It is highly unlikely that these changes are caused by an enzymatic activity of BA0331, which according to the sequence and structure of the homologous BA0330 does not modify tri- and tetrapeptides in the PG. Such function is rather associated with ld-carboxypeptidases, which trim the tetrapeptides to tripeptides. Several ld-carboxypeptidase genes have been identified (55–57), and recently, the structure of the LdcB ld-carboxypeptidase from *B. subtilis*, which is unrelated to those of BA0330 and BA0331, has been elucidated (58). Further experiments are required to test whether BA0331 is involved in regulating ld-carboxypeptidase activity.

##### Both BA0330 and BA0331 Have a Structural Role in Cell Wall Stability

The Δ*ba0330* mutant strain showed a reduced autolysis rate compared with wild type (Fig. 5*B*), suggesting an effect of BA0330 on the endogenous PG hydrolases. Reduced lysis is a property of mutations of most members of deacetylases' family, because from the PDA mutants of *B. anthracis* examined so far, only Δ*ba1977* lysed with rates comparable with that of the wild type (15). TEM revealed partial detachment of PG from the membrane of Δ*ba0330* mutant cells during both the exponential and stationary phase of growth (Fig. 6*A*) suggesting a structural role for BA0330, possibly stabilizing the interaction between the membrane and PG. Similar structural roles have been reported for lipoproteins anchored to the outer membrane of Gram-negative bacteria, which interact covalently or noncovalently with PG (59). Although it has been proposed that lipoproteins of Gram-positive bacteria could also have equivalent structural roles (7), to the best of our knowledge this is the first report of a lipoprotein from a Gram-positive bacterium involved in interactions with PG, probably by reinforcing the anchoring of PG on the membrane. In support of this role, GFP-BA0330 was distributed along the cell membrane and was slightly enhanced at division sites (Fig. 8*B*). Furthermore, growth of Δ*ba0330* was strongly impaired at increasing concentrations of NaCl, especially above 3.5% NaCl, where the wild-type strain was only slightly affected (Fig. 7), indicating that BA0330 is required to adapt to growth at high osmolarity. Although several *Bacillus* species and *Bacillus-*related genera are halophilic (60), to the best of our knowledge the response of *B. anthracis* to NaCl stress has not been reported. Upon exposure to high NaCl concentration, the closely related *B. cereus* (61) induces a protective response that includes the expression of proteins required for outgrowth (62).

The Δ*ba0331* mutant was not sensitive to lysozyme (Fig. 5*A*) and lysed similarly to wild type (Fig. 5*B*). Interestingly, the cells had an abnormal cell shape with variable cell diameter. Although the mechanism is not yet known, BA0331 could either have a stabilizing role at the lateral wall or be involved with guiding cell elongation (63). Intriguingly, GFP-BA0331 localized differently from BA0330 and in discrete patches distributed around the cell periphery in exponentially growing cells (Fig. 8*B*). Whether BA0331 participates in the construction of a normally shaped cell wall as a component of the cell elongation machinery needs to be addressed in future studies. A structural role of BA0331 is supported by a virtually complete detachment of the membrane from the cell wall in stationary cells of the double mutant Δ*ba0330/*Δ*ba0331*, much more than in the single Δ*ba0330* mutant, indicating that both gene products contribute to maintaining cell wall integrity (Fig. 6*A*).

To determine whether the morphological alterations of the Δ*ba0330* and Δ*ba0331* mutants are due to the putative de-*N*-acetylase enzymatic activity, we expressed protein versions lacking the catalytic aspartic acid residue, BA0330 (D205A) and BA0331 (D212A). Remarkably, these inactive BA0330 and BA0331 versions fully complemented the morphological aberrancies of the respective mutants, indicating that the phenotypes are not caused by the lack of enzymatic activity and supporting our conclusion that the main function of the two proteins is nonenzymatic (Fig. 9*B*).

##### Concluding Remarks

In this study, we present experimental support for novel functions of two lipoproteins, putative PDAs from *B. anthracis* in the adaptation of the bacterium under salt stress (BA0330), in cell shape maintenance (BA0331), and structural integrity of the bacterial envelope (BA0330 and BA0331). Further characterization of this system should provide a better understanding of the mechanisms by which lipoproteins maintain cell wall integrity in Gram-positive bacteria and of how bacteria generate and maintain different shapes, a fundamental question in cell biology.

## Supplementary Material

Supplemental Data

## Footnotes

4 E. Eliopoulos, personal communication.

## Footnotes

3 The abbreviations used are:

PG: peptidoglycan
PDA: polysaccharide deacetylase
TEM: transmission electron microscopy
BHI: brain-heart infusion
Spc: spectinomycin
SEM: scanning electron microscopy
Fn3: fibronectin type 3.

## Note Added in Proof

Supplemental Table 1 was missing in the version of this article that was published as a Paper in Press on March 30, 2015. The supplemental table is now available.
