# Supplementary material for: Two Putative Polysaccharide Deacetylases Are Required for Osmotic Stability and Cell Shape Maintenance in Bacillus anthracis
Source: J Biol Chem. 2015 Mar 30;290(21):13465–78. doi: 10.1074/jbc.M115.640029 (PMC4505593; doi:10.1074/jbc.M115.640029)
Supplement: Supplemental Data [file supp_290_21_13465__index.html]

Two Putative Polysaccharide Deacetylases Are Required for Osmotic Stability and Cell Shape Maintenance in Bacillus anthracis — Putative Polysaccharide Deacetylases from Bacillus anthracis — Supplemental Data 

# Two Putative Polysaccharide Deacetylases Are Required for Osmotic Stability and Cell Shape Maintenance in *Bacillus anthracis*

## Supplemental Data

**Files in this Data Supplement:**

- Supplemental Table
