# Supplementary material for: Two Putative Polysaccharide Deacetylases Are Required for Osmotic Stability and Cell Shape Maintenance in Bacillus anthracis
Source: J Biol Chem. 2015 Mar 30;290(21):13465–78. doi: 10.1074/jbc.M115.640029 (PMC4505593; doi:10.1074/jbc.M115.640029)
Supplement: Supplemental Data [file supp_M115.640029_supplementary_table.doc]

**SUPPLEMENTAL ΙNFORMATION**

**Two putative polysaccharide deacetylases are required for osmotic stability and cell shape maintenance in *Bacillus anthracis***

Sofia Arnaouteli^1^, Petros Giastas^2^, Athina Andreou^3^, Mary Tzanodaskalaki^4^, Christine Aldridge^5^, Socrates J. Tzartos^2,6^, Waldemar Vollmer^5^, Elias Eliopoulos^3^, Vassilis Bouriotis^1,4^

1. Department of Biology, Enzyme Biotechnology Group, University of Crete, PO Box 2208, Vasilika Vouton, 714 09, Heraklion, Crete, Greece.

2. Department of Neurobiology, Hellenic Pasteur Institute, Vasilissis Sofias 127, 11521, Athens, Greece.

3. Laboratory of Genetics, Department of Biotechnology, Agricultural University of Athens, Iera Odos 75, 11855, Athens, Greece.

4. Institute of Molecular Biology and Biotechnology, 70013, Heraklion, Crete, Greece.

5. Institute for Cell and Molecular Biosciences, The Centre for Bacterial Cell Biology,

Newcastle University, Newcastle upon Tyne, NE2 4AX, United Kingdom.

6. Department of Pharmacy, University of Patras, 26504 Rio, Patra, Greece.

Running title: Putative polysaccharide deacetylases from *Bacillus anthracis*.

Address correspondence to:

Vassilis Bouriotis, Department of Biology, Enzyme Biotechnology Group, University of Crete, PO Box 2208, Vasilika Vouton, 714 09, Heraklion, Crete, Greece. Tel: +302810394375; Fax: +2810394055. E-mail: bouriotis@biology.uoc.gr

Keywords: polysaccharide deacetylases, lipoprotein, *Bacillus anthracis*, cell shape osmotic stability.

**Supporting table**

**Table S1.** List of oligonucleotides used in the present study.

| oligonucleotides | Sequence (5’→3’) | Source or reference |
| --- | --- | --- |
| Construction of *B. anthracis* mutants |  |  |
| SPC-H^+1c^ | TTTTAGTTGACTTCATTTATATTTTCCTCCTTAGCCTAATTGAGAGAAGTTTCTAT | [34] |
| SPC-H^+2c^ | TTTTAGTTGACTCATTTATATTTTCCTCCTTAGCCTAATTGAGAGAAGTTTCTAT | [34] |
| SPC-H^+3c^ | TTTTAGTTGACCATTTATATTTTCCTCCTTAGCCTAATTGAGAGAAGTTTCTAT | [34] |
| ba0330up5 | AAATGAGATAGACAAACCAA | this study |
| ba0330up3 | TCCCCCGGGACGCCAAATTTTATATTGTA | this study |
| ba0331up5 | TCGTTTGTTCGTTATTAACA | this study |
| ba0331up3 | TCCCCCGGGGTAATAACTCCTTGCGTTAA | this study |
| ba0330/0331up5 | AAATGAGATAGACAAACCAA | this study |
| ba0330/0331up3 | TCCCCCGGGGTAATAACTCCTTGCGTTAA | this study |
| ba0330down5 | TCCCCCGGGGCATACACCATACGACGA | this study |
| ba0330down3 | TGTTACCTGCAAATGCTAAC | this study |
| ba0331down5 | TCCCCCGGGTGAGTTCGCAGTAACTACT | this study |
| ba0331down3 | TCCTTCAGCATCAACATTAT | this study |
| ba0330/0331down5 | TCCCCCGGGGCATACACCATACGACGA | this study |
| ba0330/0331down3 | TCCTTCAGCATCAACATTAT | this study |
| PR-5 | AATTGGGCCCGACGTCGCATG | this study |
| PR-3 | GAGCTCTCCCATATGGTCGAC | this study |
| Spec-40 | GGAGAGTGTGATGATAAGTGGG | this study |
| Spec-30 | CGCTGTTAATGCGTAAACCACC | this study |
| Construction of *gfp*-fusions |  |  |
| gfpmut1frw | CATGCATGCATGAGTAAAGGAGAAGAACT | this study |
| gfpmut1rev | GAAGATCTCTATTTGTATAGTTCATCCAT | this study |
| ba0330frw | GGGGTACCATGAGAAAATACGCAGCAAT | this study |
| ba0330rev | CATGCATGCGGCCCGGGCCCGTTTAATCGAAGAAGCAAATTG | this study |
| ba0331frw | GGGGTACCATGAAAAAGTATACATATATCG | this study |
| ba0331rev | CATGCATGCGGCCCGGGCCCGCTTTATAAGAGATATGAATTTTT | this study |
| Cloning for complementation |  |  |
| ba0330frw | GGGGTACCATGAGAAAATACGCAGCAAT | this study |
| ba0330rev | GAAGATCTTTATTTAATCGAAGAAGCAAAT | this study |
| ba0331frw | GGGGTACCATGAAAAAGTATACATATATCG | this study |
| ba0331rev | GAAGATCTTTACTTTATAAGAGATATGAATT | this study |
| Cloning for expression |  |  |
| bα0330frw | ATGAGCAATGTAAGCCAGG | this study |
| bα0330rev | CCGCTCGAGTTATTTAATCGAAGAAGCAAATT | this study |
| bα0331frw | ATGAGTGATAAACAAATAC | this study |
| bα0331rev | CCGCTCGAGTTACTTTATAAGAGATATGAA | this study |
| Cloning for site-directed mutagenesis | (engineered codons are underlined) |  |
| ba0330D205Afrw | TTTGTTACATTTGCTGATGGTATGAAAAATAATATG | this study |
| ba0330D205Arev | CATATTATTTTTCATACCATCAGCAAATGTAACAAA | this study |
| ba0331D212Afrw | TTCATAACAATGGCTGATGGTCGAAAAAATAATATG | this study |
| ba0331D212Arev | CATATTATTTTTTCGACCATCAGCCATTGTTATGAA | this study |
